# Relations between tactile sensitivity of the finger, arm, and cheek skin over the lifespan showing decline only on the finger

**DOI:** 10.3389/fnagi.2024.1387136

**Published:** 2024-07-02

**Authors:** Léonard Samain-Aupic, Mariama Dione, Edith Ribot-Ciscar, Rochelle Ackerley, Jean-Marc Aimonetti

**Affiliations:** Aix Marseille Univ, CNRS, CRPN (Centre de Recherche en Psychologie et Neurosciences - UMR 7077), Marseille, France

**Keywords:** glabrous skin, hairy skin, aging, discriminative touch, tactile sensitivity

## Abstract

Touch sensitivity generally declines with age, contributing to loss of manual dexterity and tactile function. We investigated how touch changes over the lifespan, using different tests and on three body sites. We used a classical test of force detection sensitivity, where calibrated monofilaments were applied passively to the right index finger pad, forearm, and cheek. In addition, at the index, we used an active touch spatial discrimination task, developed by our group. Spatial discrimination was estimated through participants' ability to evaluate the distance between parallel bands printed on acrylic plates. Data were collected from 96 healthy women, aged 20–75 years. Force detection and tactile spatial discrimination on the index deteriorated significantly with age; however, no change was found for tactile detection on the forearm or cheek. Tactile detection on the cheek remained remarkably highly sensitive throughout life. There was a significant positive relationship between force detection and spatial discrimination on the index. Further, force detection on the forearm was significantly associated with detection on the index and cheek. Our results suggest a decrease in touch perception with age on the index finger pad, yet a preservation of tactile sensitivity in hairy skin. This opens discussion about the impact of daily activities upon the glabrous hand skin and on the function of hairs in tactile sensitivity. We highlight the need for new methods in evaluating tactile sensitivity on hairy skin.

## 1 Introduction

Aging is associated with a decline in most sensory functions. While aging of the auditory and visual systems is more readily measured, tactile aging has been less studied, in part due to its heterogeneity (McIntyre et al., 2021). The functional consequences of tactile aging nevertheless cause numerous impairments, from decreased efficiency in haptic object manipulation using the hands (Wickremaratchi and Llewelyn, 2006) to deficits in postural feedback from the feet, leading to an increased risk of falls in older adults (Soriano et al., 2007). However, the effect of aging is often body-site dependent, such as sunlight damage on facial skin (Shin et al., 2023), and it can be variable between individuals, for example, highly preserved tactile function on the glabrous hand skin in some older people (Skedung et al., 2018). The foot skin is also particularly susceptible to aging (Stevens and Choo, 1996). Many touch tests can be applied all over the body, where differences in sensitivity can be seen due to the type and density of mechanoreceptors present (Corniani and Saal, 2020). Touch tests can measure many different aspects of tactile perception, thus give different results that need to be interpreted carefully. Further, the accuracy of such tests depends on the sensitivity of the test, potential biases, and its reliable application (Bell-Krotoski et al., 1993; Tong et al., 2013). Thus, simple and reliable touch tests are required to better understand tactile function, especially with age and in disorders.

Skin is the barrier that segregates our body from the external environment and endures both intrinsic and extrinsic factors in aging (Krutmann et al., 2021; Shin et al., 2023). Intrinsic aging stems from bodily physiological processes (e.g., decreasing blood flow, hormonal changes) that leads to thinner, drier skin, fine wrinkles, and gradual dermal atrophy. Extrinsic aging is caused by external factors such as wear-and-tear, air pollution, smoking, and sun exposure, resulting in coarse wrinkles and a loss of elasticity. Both intrinsic and extrinsic factors impede skin repair and the first signs of skin aging appear around the age of 30, when collagen and elastin synthesis decrease (Lephart, 2016). These changes in skin properties may all contribute to the decline of tactile sensitivity to varying degrees (Lévêque et al., 2000; Skedung et al., 2018; Aimonetti et al., 2019), as well as the potential for cognitive decline affecting tactile perception and processing (Löffler et al., 2024).

Among the mechanisms responsible for tactile aging, much attention has been paid to changes in the peripheral nervous system. The number of nerve fibers in the dermis and epidermis decreases with age, affecting body parts differently (Verdú et al., 2000; Besné et al., 2002). Mechanotransduction may be also affected. Skin deformation is translated into action potentials in afferent receptors via Piezo2 stretch-sensitive ion channels (Ranade et al., 2014). In mice, a genetic deletion of Merkel cells and associated mechanosensitive Piezo2 channels in the skin is sufficient to produce allokinesis (evoked itch by innocuous touch), known to occur in aged and dry skin; however, very little is known in humans (Feng et al., 2018). In the glabrous skin, the loss of receptor endings has been widely documented, where the density of Merkel cells and Meissner corpuscles decreases with age (Cauna, 1965; Iwasaki et al., 2003). A loss of Meissner corpuscles has been associated with a lower number of Piezo2 channels (García-Piqueras et al., 2019) and lower tactile perception performance with age (Skedung et al., 2018).

Less is known about the specific innervation of the hairy skin, which covers the vast majority of the body and is highly heterogenous. Human microneurography investigations on nerves that innervate hairy skin have mostly focused on proprioceptive afferents and on C afferents (Corniani and Saal, 2020) and aging has been associated with changes in C-fiber activity (Namer et al., 2009). Further work has documented a reduced epidermal innervation with age (Decorps et al., 2014).

Changes in the central nervous system with age are now well-recognized. Aging is associated with a loss of neurons and myelin in the brain, which accelerates after 70 years of age (Salat et al., 2005; McIntyre et al., 2021). This is accompanied by a decrease in cerebral blood flow, which contributes to slower response latencies, as found in rodents (Godde et al., 2002). All these factors may contribute to a decrease in neural function accompanied by cognitive decline, which together further impairs tactile processing.

Considering the diversity and variability found in human tactile perception, we aimed to evaluate tactile sensitivity in terms of tactile detection threshold in glabrous (index finger tip) and hairy (forearm and cheek) skin in women aged from 20 to 75 years. Tactile spatial discrimination was also evaluated through an active touch task using the index finger pad in the same participants. We hypothesized a deterioration in tactile sensitivity at the glabrous fingertip in aging, with the potential for this at the hairy skin sites. We sought to explore the relationships between values obtained with the different tests to determine whether similarities exist in aging impairments between the various skin areas tested.

## 2 Materials and method

### 2.1 Participants

Ninety six healthy (five left-handed) women aged between 20 and 75 years old participated in the study. They were self-reported free of neurological, psychiatric, dermatological disorders, or clinically significant peripheral neuropathy. These participants were recruited over three experiments that shared similar protocols. Forty three participants aged between 40 and 60 years old were included from a first study (Samain-Aupic et al., 2023). Forty two participants were included from a second study composed of 2 groups aged between 20 and 30 years old and 65 and 75 years old (Dione et al., 2023). Data from these two experiments were extracted from the baseline conditions, as both studies aimed to test the effects of applying hydrating agents to the skin. To complete the sample over the full age range, a third group of 11 participants aged between 30 and 40 years old were specifically added in the present study. The work was carried out in accordance with the Declaration of Helsinki, apart from pre-registration in a database, and was approved by an ethical committee (Comité de protection des personnes Est-III). All participants gave their written informed consent.

### 2.2 Procedures

All the experiments took place in the same quiet room with a constant temperature of 21°C. Participants were asked to sit comfortably in a chair, close their eyes and wear noise canceling headphones (Bose QuietComfort 25, Framingham, MA), to avoid visual and auditory cues during the experiments. Three skin sites were tested, namely the index finger, forearm, and cheek on the right side. Tactile detection thresholds were measured at all sites using calibrated monofilaments and an additional test of tactile spatial discrimination, using plates with different sized grooved bands, was also performed on the finger skin. For further details, see Aimonetti et al. (2019), Dione et al. (2023), and Samain-Aupic et al. (2023).

#### 2.2.1 Tactile detection test

Tactile detection thresholds were measured using a range of 13 calibrated monofilaments (range: 78, 59, 39, 20, 14, 10, 6, 4, 1.6, 0.7, 0.4, 0.2, and 0.08 mN) (Ugo Basile). They were applied to the index fingertip, the ventral forearm at 10 cm distal from the wrist, and in the middle of the cheek. The order of areas tested was pseudo-randomized and counter-balanced between participants. Participants were familiarized with a short pre-test procedure where the 40 mN filament was applied on the index and they had to say if they felt the stimulation (which they all did).

During the test phase, participants had to close their eyes and said “top” when they felt a stimulation on the area tested. A staircase procedure was performed to obtain a tactile detection threshold. For each monofilament, three applications were performed. The experiment started with the 40 mN monofilament. If three applications were felt, the next monofilament tested decreased by two monofilament force levels (14 mN). If all stimulations were felt again, the next monofilament increased by one force level (20 mN). This procedure was repeated until the participant made one error. When an error was made, the next monofilament force level increased by one until the participants felt the three applications. The test ended when two errors were made and the detection threshold was noted as the preceding monofilament level.

#### 2.2.2 Spatial discrimination test

The spatial discrimination test was performed on the index finger pad of the right hand and participants were asked to explore plates with a single downward movement of the finger. The participant was instructed to make a smooth movement over the plate from top to bottom, at about 20 mm/s (Vega-Bermudez et al., 1991), which the experimenter ensured they could do in a short pre-test familiarization. The plates had inter-band groove spacings that varied from 3.6 mm to 6 mm. Eleven plates were tested and the middle plate with 4.8 mm inter-band-groove was assigned as the reference plate. The others test plates varied by 0.2 mm from the reference except for the two extreme plates where the inter-band groove changed by 0.4 mm (Aimonetti et al., 2019). A trial consisted of the participants exploring two different plates, where one was always the reference. They had to say which plate had the larger inter-band spacing. The order of presentation of test plate order was pseudo-randomized, as was the location (left or right) of the reference plate.

For the first group of participants, the test plates were compared 15 times with the reference except for the two extreme test plates, which were compared six times with the reference. A total of 132 comparisons were presented for each participant in this group. Due to time constraints in the second and third groups, the test was performed with eight comparisons of test plates, except for the two extreme plates that were compared four times with the reference. In these two participant groups, a total of 72 comparisons were presented, and the instructions remained the same. The responses were expressed in the percentage of responses when participants said that the test plate had a larger spacing than the reference.

### 2.3 Statistical analyses

Statistical analyses were conducted using Prism (version 8; GraphPad). The tactile detection test data were log transformed, where values were in the range between −2 that corresponded to 0.08 mN and 0.3 that corresponded to 20 mN. This was to normalize the scale and lower values indicated better performance. The actual monofilament detection forces are shown in the figures on a log scale, for ease of interpretation.

For the spatial discrimination test, psychophysical curves were obtained and difference thresholds were calculated with the Palamedes toolbox (Prins and Kingdom, 2018) in MATLAB (The Mathworks). The difference threshold corresponded to the minimum inter-band spacing (in mm) to perceive a difference and was obtained by subtracting the projection from 75% to 50% of correct answers on the y-axis onto the x-axis inter-band spacing on the psychophysical curve for each participant.

Linear regressions were performed between age and the results for each of the four tactile tests. To compare performance between tests, we also carried out linear regression between each test. All individual data points are plotted in the figures and upper and lower 95% confidence intervals (CI) of the mean are given where appropriate.

## 3 Results

### 3.1 Touch test performance with age

**Figure 1:**
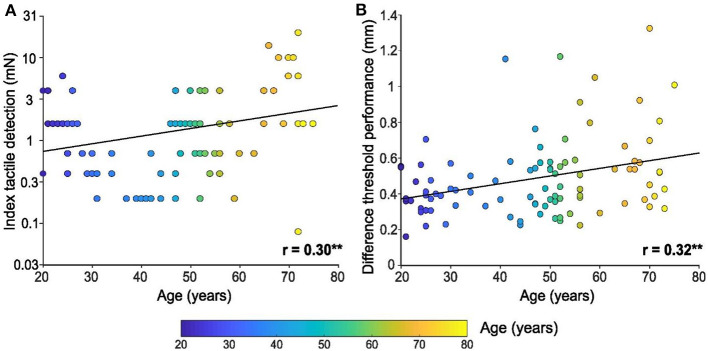
Tactile detection and spatial discrimination on the index finger pad with age. **(A)** Linear regression of tactile detection performance of the index in mN (log scale), according to age. **(B)** Linear regression of spatial discrimination performance (in mm), according to age. Individual results (*n* = 96) are shown as a function of the age of the participant (color bar), ** *p* < 0.01.

Regarding tactile detection on the index finger using calibrated monofilaments, we found that the detection threshold increased linearly with age, thus showing a deterioration of tactile capacity over the lifespan [*F*_(1, 94)_ = 9.6, *p* = 0.003, *r* = 0.30, Figure 1A]. The ten youngest participants (mean = 22 years ± 1.5 SD) had a mean index tactile detection threshold of 2.4 mN ± 0.5 SEM (95% CI 1.1–3.6 mN), whereas the ten oldest participants (mean = 72 years ± 1.5 SD) had a threshold of 5.8 mN ± 1.9 SEM (95% CI 1.5–10 mN). Intriguingly, the participant with the lowest threshold (0.08 mN, best performer) was actually 72 years old, whereas the participant with the highest threshold (20 mN, worst performer) was also 72 years old, showing the variability that can occur in touch perception with age.

Spatial discrimination tests were also performed using the index finger and a significant linear relationship between performance and age was found [*F*_(1, 94)_ = 11.0, *p* = 0.001, *r* = 0.32, Figure 1B], where spatial discrimination capacity worsened with age. The minimum threshold for tactile spatial discrimination obtained was 0.16 mm for a 21-year-old participant, where the mean threshold for the 10 youngest participants was 0.38 mm ± 0.04 SEM. The maximum threshold obtained was 1.3 mm for a 70-year-old participant, where the mean threshold for the 10 oldest participants was 0.62 mm ± 0.01 SEM.

**Figure 2:**
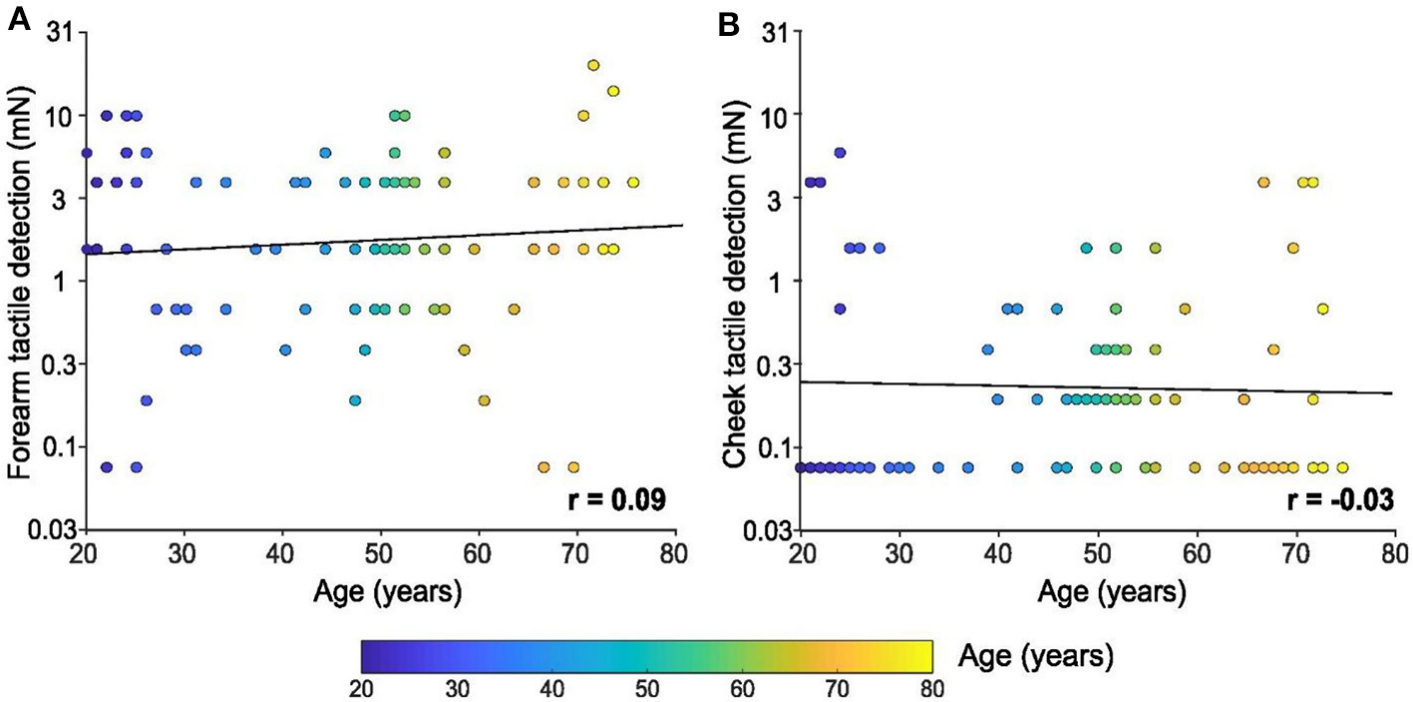
Tactile detection on the forearm and cheek with age. Linear regression of tactile detection performance of the **(A)** forearm and **(B)** cheek in mN (log scale) according to age. Individual results (*n* = 96) are shown as a function of the age of the participant (color bar). There was no significant relationship between either skin site and the age of the participants.

The tactile tests on the index finger clearly showed decreases in touch capacity with age. Tactile detection tests using calibrated monofilaments were also carried out on the forearm and cheek, yet conversely, no significant linear relationship was found between tactile detection and age for either skin site. Figure 2A shows the forearm results [*F*_(1, 94)_ = 0.7, *p* = 0.403, *r* = 0.09] and Figure 2B the cheek results [*F*_(1, 94)_ = 0.1, *p* = 0.780, *r* = −0.03], where it can be seen that the cheek was highly sensitive to touch throughout the lifespan, with many participants achieving the lowest detection level possible for our test (0.08 mN). Inspecting the ten youngest participants again, we find a mean forearm tactile detection threshold of 4.7 mN ± 1.1 SEM (95% CI 2.3–7.1 mN), whereas the ten oldest participants had a threshold of 5.6 mN ± 2.0 SEM (95% CI 1.2–10 mN). Conversely, we found a mean cheek tactile detection threshold for the ten youngest participants of 0.9 mN ± 0.5 SEM (95% CI 0.2–2.1 mN), whereas the ten oldest participants had a threshold of 1.1 mN ± 0.5 SEM (95% CI 0.05–2.2 mN).

### 3.2 Comparisons of performance between touch tests

**Figure 3:**
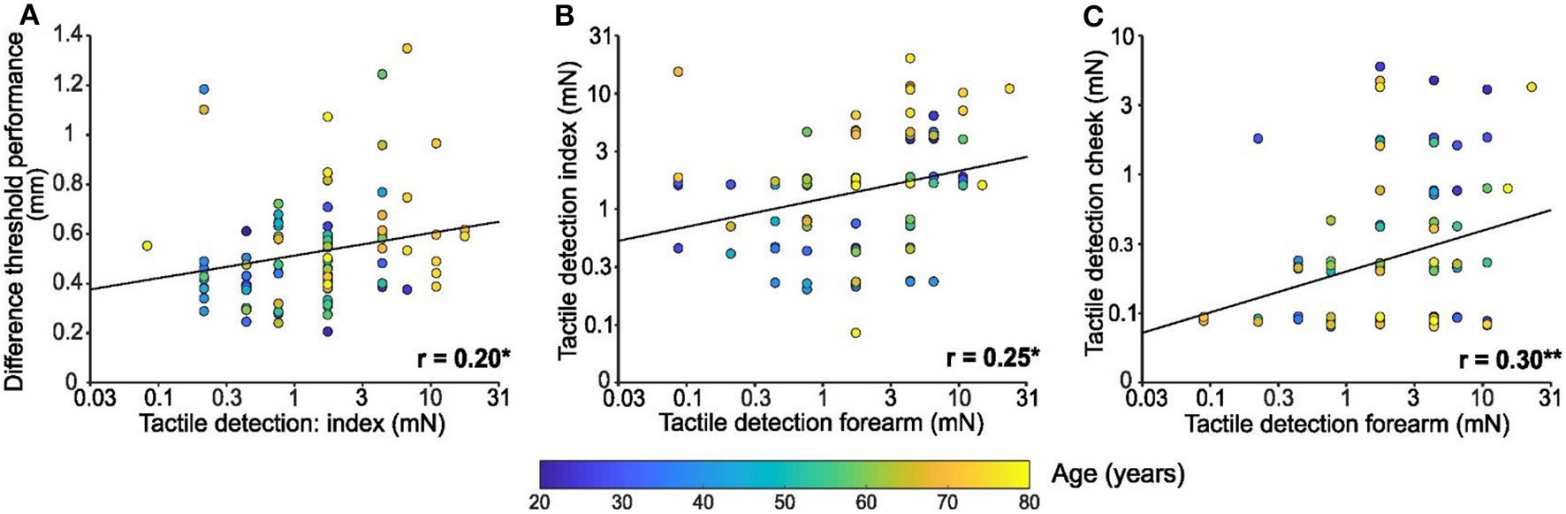
Comparisons between tactile test performances. **(A)** Linear regression of spatial discrimination performance in mm according to tactile detection performance of the index in mN (log scale). **(B)** Linear regression of tactile detection performance of the index in mN (log scale) according to tactile detection performance of the forearm in mN (log scale). **(C)** Linear regression of tactile detection performance of the cheek in mN (log scale) according to tactile detection performance of the forearm in mN (log scale). Individual results (*n* = 96) were colored in function of the age of the participant as shown by the color bar, * *p* < 0.05, ** *p* < 0.01.

As well as investigating the change in tactile perception with age, we compared performance between our tests. Regarding the index finger, performance on the spatial discrimination test was positively associated with tactile detection performance [*F*_(1, 94)_ = 4.1, *p* = 0.046, *r* = 0.20, Figure 3A]. However, no relationship was found between finger spatial discrimination capacity and tactile detection for the other areas tested was found (forearm: *p* = 0.279, cheek: *p* = 0.436; not shown).

Comparing the performance on tactile detection tests between sites, index detection was significantly positively associated with forearm detection [*F*_(1, 94)_ = 6.5, *p* = 0.013, *r* = 0.25; Figure 3B]. Similarly, forearm detection was significantly positively associated with cheek detection [*F*_(1, 94)_ = 9.03, *p* = 0.003, *r* = 0.30; Figure 3C]. However, there was no significant relationship between tactile detection capacity on the index and cheek (*p* = 0.74; not shown).

## 4 Discussion

Presently, we found that tactile sensitivity deteriorated with age on the glabrous finger skin, but not on the forearm or cheek. This decrease in finger tactile capacity with age was found in both the tactile detection and spatial discrimination tests. Further, we found that tactile detection on the forearm was related to detection capacity on the finger and cheek. We discuss why the finger may be susceptible to aging and why touch perception on the hairy skin, especially that of the cheek, appears to be conserved with age. Our regressions showed a clear link between touch tests on the finger, implying that different finger tactile capacities are affected with age. However, we also found other relationships between touch detection at different body sites, unconnected with age, demonstrating that some people may have increased touch capacities over others. It is also evident that better-adapted tests are needed to explore touch over the body, as we consistently reached the limit of the tactile detection test on the cheek.

The differences in the impact of aging on different skin areas may be due to numerous intrinsic factors. Firstly, human skin is highly heterogenous over the body, where glabrous skin and hairy skin are fundamentally different and may age in different ways. This may include changes in blood flow (Stevens and Choo, 1996) and decreased output to the skin of the sympathetic nervous system (Mano et al., 2006), including decreased sweating that may lower skin moisture levels, contributing to tactile perception deterioration (Dione et al., 2023). Glabrous skin has a thicker epidermis and has a generally high density of mechanoreceptors, as well as containing Meissner corpuscles (Ackerley, 2023). It is known that mechanoreceptor organ and axonal loss contributes to touch degradation on glabrous skin with age (García-Piqueras et al., 2019). Hairy skin is usually assumed to have a lower density of mechanoreceptors, although this is a generalization, as the face is densely innervated (Nordin and Hagbarth, 1989). Although hairy skin is believed to lack Meissner corpuscles, it has an additional source of information: hairs. Hairs have a protective function for the skin, to decrease heat loss (Romanovsky, 2014), but also act as an antenna to transmit mechanical stimuli, including at very low forces, such as an air flow. Body hair has been found to decrease throughout life, although especially on the legs (Melick and Taft, 1959). In our study, the monofilament was never directly applied on visible thick hairs, but could have very well touched thin hairs, activating mechanoreceptors and thus facilitating detection. We decided not to remove hairs in our paradigm, as the hair stimulation is an essential component of touch. This may explain the superior performance observed at the cheek, regardless of the age of participants. Previous work has demonstrated the high density of hairs on the face, with a typical density of between 10–30 times more than all other investigated skin sites (Otberg et al., 2004).

Another explanation for differences between glabrous hand skin and the hairy skin could be due to extrinsic factors. The hands might be particularly affected, as they are usually unprotected and are used extensively throughout the day. Activities such as hand-washing and the use of cleaning products may further impact hand tactile sensitivity (Slaughter et al., 2019). Similarly, hands are often exposed to extreme temperatures that can reduce manual dexterity, particularly in old people (Tajmir et al., 2013). We should also consider experience, for example, professional practices requiring fine manual skills may help prevent tactile decline (Zamorano et al., 2015; Godde et al., 2018). Conversely, the arm skin is often protected by clothes and less exposed to external elements, potentially preserving it with age. We did not presently test the hairy skin on the dorsum of the hand, but this would provide a close control to compare to the aging of the glabrous hand skin. The facial skin is different still: it is the most exposed, at least to sun radiation (Bonté et al., 2019), yet sensitivity is preserved (Stevens and Choo, 1996). These factors may explain why tactile impairment with aging is highly variable, where some people are more sensitive in touch generally, as found in our results, and why a proportion of the elderly have maintained tactile capacity (Skedung et al., 2018).

It is likely that people whose professional activity requires fine manual dexterity may care more for their skin, such as using moisturizer. The contribution of skin hydration to tactile sensitivity has been shown in different studies, where touch detection thresholds and spatial discrimination improve immediately after applying a moisturizing cream (Lévêque et al., 2000; Bowden and McNulty, 2013; Skedung et al., 2018). Similarly, tactile spatial discrimination significantly improved after 1 month of cream application (Aimonetti et al., 2019). It seems thus that preserving skin properties, such as hydration and elasticity, by daily application of cosmetic products may help sustain and even ameliorate somatosensory function that is associated with declining tactile capacity with age. Another explanation lies in a difference in neuronal decline associated with receptor sensitivity or density and/or signal transmission. For example, the best-performing aged individuals on tactile tests have a greater density of Meissner corpuscles (Skedung et al., 2018), while Meissner corpuscles quality generally declines with aging (García-Piqueras et al., 2020). Finally, we also need to consider body size, with differences being more marked at the extremities. Any participant with large hands could be less sensitive than another participant of the same age with smaller hands, likely because the number of nerve fibers remains relatively stable overall with body size (Peters et al., 2009). Thus, tactile decline may be more evident in someone later in life who has larger hands (Creigh et al., 2022). All these parameters combined would explain the high inter-individual variability in performance, whether in terms of sensitivity to pressure or spatial discrimination, a variability that increases with age.

The present results lead us to suggest that tactile sensitivity might remain relatively constant throughout adulthood on the cheek and forearm, for various possible reasons. However, we also need to consider whether we were unable to detect an impairment because of the test used. This was less likely for the forearm, but the participants often achieved the lowest detection level for the cheek. Tactile detection using monofilaments tests the basic sense of touch, i.e., whether a mechanical stimulus was detected or not, which is different to whether this could be accurately located (point localization) or distinguished from another stimulus (e.g., two-point discrimination) (Bell-Krotoski et al., 1993). As we often reached the lowest force, thinnest monofilaments, this means that we could not measure the true level of detection. Lighter force monofilaments would be ideal to use in the future. However, very fine monofilaments are difficult to apply, being so thin that they often move over the skin rather than bending, hence being less reliable in-use. A more precise test is required for detection, which could take the form of monofilaments made from different materials.

The simple monofilament touch detection test probes the basic recognition of a touch stimulus, whereas tactile localization and discrimination generally require more effort, including increased cognition (e.g., attention, processing capacity). Two-point discrimination has been well-criticized for its biases and unreliability (Bell-Krotoski et al., 1993; Tong et al., 2013), although there are related spatial acuity tests that can overcome these issues. For example, JVP Domes can be used where the participant discriminates between horizontal and vertical lines (Wong et al., 2011) or adapted localization tests measuring spatial acuity (Long et al., 2022). An advantage of our tactile spatial discrimination approach is that it uses active touch, which is a naturalistic way of interacting with surfaces. However, it is only suited to parts of the body used for such exploratory, active touch. The detection of other stimuli, such as air flow or liquids, may overcome the limits of existing tests, permitting the testing of very low force mechanosensation capacity, regardless of the body part explored.

Overall, developing finer and more reliable touch tests would be useful both for research and clinical purposes. Tests that are non-invasive and non-painful, yet precise, may help in the earlier identification of somatosensory issues, where treatment could begin earlier, leading to better outcomes. As well as the development of better touch tests, especially for use on sensitive hairy skin areas, future work should expand the age range that we studied (i.e., < 20 and more than 75 years' old) and add more participants, including men. This would allow the investigation of the inherent variability of the population and the exact relationship of tactile sensitivity with age. Further, the contribution of body hair of different thickness to tactile sensitivity would also be of interest to explore. It may be that hairy skin tactile capacity is preserved with age, thus this could provide a means for conveying important discriminative and affective touch information in the elderly, providing an effective way to communicate and convey messages and sentiments.

## Data availability statement

The datasets presented in this study can be found in online repositories. The raw data from this study is available at https://osf.io/879kb.

## Ethics statement

The studies involving humans were approved by the Comité de protection des personnes Est-III. The studies were conducted in accordance with the local legislation and institutional requirements. The participants provided their written informed consent to participate in this study.

## Author contributions

LS-A: Conceptualization, Formal analysis, Investigation, Methodology, Validation, Visualization, Writing—original draft, Writing—review & editing. MD: Methodology, Validation, Writing—original draft, Writing—review & editing. ER-C: Writing—original draft, Writing—review & editing. RA: Conceptualization, Data curation, Formal analysis, Funding acquisition, Investigation, Methodology, Project administration, Resources, Supervision, Validation, Visualization, Writing—original draft, Writing—review & editing. J-MA: Conceptualization, Funding acquisition, Investigation, Methodology, Project administration, Resources, Supervision, Validation, Writing—original draft, Writing—review & editing.
